# Comparing predictions among competing risks models with rare events: application to KNOW-CKD study—a multicentre cohort study of chronic kidney disease

**DOI:** 10.1038/s41598-023-40570-2

**Published:** 2023-08-16

**Authors:** Jayoun Kim, Soohyeon Lee, Ji Hye Kim, Dha Woon Im, Donghwan Lee, Kook-Hwan Oh

**Affiliations:** 1 Medical Research Collaborating Center, Seoul National University Hospital, Seoul, Republic of Korea; 2 Department of Statistics, Ewha Womans University, Seoul, Republic of Korea; 3 Department of Internal Medicine, Chungbuk National University Hospital, Cheongju, Korea; 4 Department of Internal Medicine, Seoul National University Hospital, Seoul, Republic of Korea

**Keywords:** Kidney diseases, Statistics

## Abstract

A prognostic model to determine an association between survival outcomes and clinical risk factors, such as the Cox model, has been developed over the past decades in the medical field. Although the data size containing subjects’ information gradually increases, the number of events is often relatively low as medical technology develops. Accordingly, poor discrimination and low predicted ability may occur between low- and high-risk groups. The main goal of this study was to evaluate the predicted probabilities with three existing competing risks models in variation with censoring rates. Three methods were illustrated and compared in a longitudinal study of a nationwide prospective cohort of patients with chronic kidney disease in Korea. The prediction accuracy and discrimination ability of the three methods were compared in terms of the Concordance index (C-index), Integrated Brier Score (IBS), and Calibration slope. In addition, we find that these methods have different performances when the effects are linear or nonlinear under various censoring rates.

## Introduction

Survival analysis has been widely used in biomedical research to investigate the effects of clinical risk factors on survival outcomes. The Cox proportional hazards model^1,2^ is a widely applied method for assessing survival outcomes during the follow-up period. Although one of the key benefits of the Cox model is that it does not assume the shape of the baseline hazard, it only considers single or first events. Meanwhile, we often encounter some cases in which patients experience multiple events over time. If the events preclude the occurrence of the event of interest, then they can be competing risks. For example, when the event of interest is a death caused by cardiovascular disease, another occurrence of death due to non-cardiovascular disease can be a competing risk^3–5^. Considering this situation, more extended statistical methods are needed to investigate the presence of competing risks.

The first approach to adapting competing risks data is the Cause-specific hazard (CS) model, which estimates the hazard of each event separately^6^. The second approach is the Fine and Gray (FG) model^7^, also known as the sub-distribution hazard model to estimate the hazards of the cumulative incidence function. Because the main difference between these two approaches is the presence or absence of competing risks in the risk set, they can yield different results^4^. Recently, Ishwaran et al.^8^ introduced the Random survival forests (RSF) which is a notable approach for application to competing risks data in a machine learning framework. The RSF is a tree-based estimation and prediction method for estimating event-specific risk factors and cumulative incidence functions non-parametrically.

Meanwhile, there may be cases in which events of interest or diseases are relatively rare as bio-medical science advances over the past decades. For instance, penile cancer is a rare disease with an incidence of only 0.1 to 0.9 per 100,000 males^9^. Because accurate and reliable results may not be obtained in the presence of rare events, more caution should be taken when considering the complexity of the data structure.

The remainder of this paper focuses on the predictive performance of three existing competing risks models with rare events. We conducted a nationwide prospective cohort study to investigate the association between clinical risk factors and adverse renal outcomes. Subsequently, we compared the predictive performance using three existing methods with real-world data. Additionally, we consider situations that exist only in linear or nonlinear effects in time-to-event outcomes and covariate terms. Subsequently, discrimination, predictive accuracy, and model calibration were evaluated using the C-index, IBS, and Calibration slope, respectively.

## Materials and methods

### Patients information and data collection

The KoreanN Cohort Study for Outcomes in Patients With Chronic Kidney Disease (KNOW-CKD) is a multi-center, patient-based cohort study launched in 2011. The objective of the KNOW-CKD is to explore the etiologic risk factors associated with the clinical course progression of CKD. A total of 2,238 eligible patients were all Koreans, aged between 20 and 75 years, with non-dialyzed CKD stages 1 from 5 based on the estimated glomerular filtration rate from serum creatinine.

Demographic characteristics and medical histories, such as smoking status, comorbidities, and cause of CKD, were obtained at baseline. Laboratory data were also collected, including hemoglobin, fasting blood sugar, uric acid, calcium, phosphorous, albumin, total cholesterol, low-density lipoprotein cholesterol, high-density lipoprotein cholesterol, and high-sensitivity C-reactive protein. The major outcomes of CKD can be progression to end-stage kidney disease (ESKD), kidney failure, death, and complications of kidney dysfunction, including cardiovascular disease (CV), anemia, and bone disease. More detailed information regarding the KNOW-CKD cohort study has been published elsewhere^10,11^.

### Risk factors

To identify possible risk factors for renal dysfunction with a competing risks model, we selected 12 covariates according to clinical consideration: age at enrollment (age), gender, baseline comorbid diseases such as coronary artery disease (CAD), diabetes mellitus (DM), cardiovascular disease (CVD), smoking status (smoking), CKD stage at the study entry (CKD_stage), body mass index (BMI), hemoglobin (HG), high-sensitivity C-reactive protein (CRP), mean arterial pressure (MAP), and low-density lipoprotein cholesterol (LDL) at baseline.

### Outcomes

In the CKD study, a CV event was defined as any first event of the following: acute myocardial infarction, unstable angina, ischemic or hemorrhagic cerebral stroke, congestive heart failure, symptomatic arrhythmia, aggravated valvular heart, pericardial disease, abdominal aortic aneurysm, and severe peripheral arterial disease. In addition, an end-stage kidney disease (ESKD) can be defined as the initiation of renal replacement therapy, such as dialysis or renal transplantation^12^.

### Methods for analyzing survival data with competing risks

#### Cause-specific hazard model

The CS model is an adaptation of the Cox model, with cause-specific hazard functions from different types of events. It treats failure from the cause of interest as events and failures from other causes as censored^13^. Let *T* and *C* denote failure and censoring times, respectively. The cause-specific hazard function at time *t* for cause $k (k=1, \cdots ,K)$ is

$$\begin{aligned} h_{k}^{CS}(t) = \lim _{\Delta \rightarrow 0}\frac{P(t < T \le t + \Delta t, K=k |T > t)}{ \Delta t}. \end{aligned}$$

Similar to the Cox model, a separate proportional hazards model with *p*-dimensional covariates for cause *k* can be defined as

$$\begin{aligned} h_{k}^{CS}(t|{{\textbf {X}}}) = h_{k0}^{CS}(t)\exp ({\varvec{\beta }}^{'}_{{\textbf {k}}}{{\textbf {X}}}), \end{aligned}$$

where $h^{CS}_{k0}(t)$ is the baseline of the cause-specific hazard function, and the exponential term illustrates the covariate effects on cause *k*. The regression coefficients $\beta _{k}$ for cause *k* can be calculated using the maximum partial likelihood estimation method as follows:

$$\begin{aligned} L(\beta _{k}) =\prod _{i} \left( \frac{{\exp (\beta {'}_{{\textbf {k}}}{{\textbf {X}}}_{i})}}{\sum _{j \in R_{i}^{CS}}{\exp (\beta {'}_{{\textbf {k}}}{{\textbf {X}}}_{j})}}\right) ^{K=k}, \end{aligned}$$

where $R_{i}^{CS}$ represents the *i*-th risk set which contains subjects who have not experienced any event and are not censored yet.

#### Fine and Gray model

The FG method^7^ is another extension of the Cox model that estimates the incidence of outcomes in a follow-up period with competing risks. Unlike the CS model, the FG model can be defined as the instantaneous rate of occurrence of each event type in subjects who are still under observation and those who have already experienced competing risks. The FG model describes the hazard function,

$$\begin{aligned} f_{k}^{FG}(t) = \lim _{\Delta \rightarrow 0}\frac{P(t< T \le t + \Delta t, K=k|T<t \cup (T<t \cap K \ne k))}{ \Delta t}. \end{aligned}$$

Analogous to the CS model, the FG model with covariates can be defined as follows:

$$\begin{aligned} h_{k}^{FG}(t|{{\textbf {X}}}) = h_{k0}^{FG}(t)\exp (\beta ^{'}_{{\textbf {k}}}{{\textbf {X}}}), \end{aligned}$$

where $h^{FG}_{k0}(t)$ is the baseline of the subdistribution hazard of cause *k*. The regression coefficient $\beta _{k}$ for cause *k* can also be calculated using the maximum partial likelihood estimation:

$$\begin{aligned} L(\beta _{k}) = \prod _{i}\left( {\frac{\exp (\beta ^{'}_{{\textbf {k}}}{{\textbf {X}}}_{i})}{\sum _{j \in R_{i}^{FG}} w_{ij} \exp (\beta ^{'}_{{\textbf {k}}}{{\textbf {X}}}_{j})}}\right) ^{K=k}, \end{aligned}$$

where $R_{i}^{FG}$ represents the *i*-th risk set, which contains subjects who have experienced competing risks ahead and are still event-free. $w_{ij}$ are subject-specific weights that reflect the incidence of competing risks.

#### Random survival forests

The RSF, first introduced by Ishwaran et al.^14^, is an extension of the machine-learning tree-based Random forests^15^. Subsequently, Ishwaran et al.^8^ proposed a fully nonparametric method for competing risks in the presence of right-censored data. As mentioned in Ishwaran et al.^8^, RSF has many useful properties. It calculates the cumulative incidence function directly in each node and provides an accurate prediction performance by aggregating individual trees in the ensemble. This allows the model to include not only linear effects but also nonlinear and interaction effects. Permutation importance was used as a measure of variable importance to identify the event-specific risk factors. A more detailed description of RSF is presented in Ishwaran et al.^8^.

RSF constructs each tree using a bootstrap sample of the original training data. We extracted .632 samples without replacement as training data in the bootstrapped sample and excluded the rest of them for out-of-bag data. Random feature selection can be applied to evaluate the split tree nodes at each node. For each tree, a cumulative hazard function was estimated using the Nelson-Aalen estimator. The survival forest is calculated by averaging the terminal node statistics in the ensemble.

Since identifying the most influential risk factors is the main interest in the medical field, it can be interesting to use Variable Importance as a means of finding a ranking of important risk factors. The RSF approach ranks covariates based on their predicted values and determines the important factors through the predicted accuracy. That is, the prediction accuracy of the test data in the current model was first calculated for each tree. Then, the prediction accuracy was also calculated on shuffled data using the same method. The differences between the original prediction accuracy and randomly permuted prediction accuracy were averaged over all trees, and they were normalized by the standard error. If a current model without the original values of a variable can provide a worse prediction, then the variables with large values are ranked as more important.

In addition to identifying important risk factors, there is one way to explain how each covariate affects the output of the model. The SHAP value is used to address each variable’s contribution to the model. The SHAP value is closely related to “Shapley values” which were first developed for the game theory method^16^. It has been widely adopted since the study by Lundberg and Lee^17^ was first published. The SHAP value translates to assigning an importance value to features, depending on their contribution to the prediction. In other words, it is calculated as the average marginal contribution of a variable value across all possible combinations^18^. Thus, the SHAP value can be the relative risk of the outcomes, meaning that high values contribute more to the predicted probability.

### Performance measures

#### Concordance index

The C-index is commonly used to identify predicted probability in a prognostic model. This is a generalization of the area under the ROC curve that considers censored data^19,20^. In randomly selected patients, those with shorter time-to-event would have higher risk scores and lower predicted time-to-event outcomes. As presented in Brentnall and Cuzick^21^, the C-index is calculated from the Wilcoxon rank-sum statistic by

$$\begin{aligned} C = P( {\hat{T}}_1 \ge {\hat{T}}_2 | T_1 \ge T_2), \end{aligned}$$

where $T_{1}$ and $T_{2}$ are survival time of the two different subjects, ${\hat{T}}_1$ and ${\hat{T}}_2$ are their predictions from a fitting method, and $I(\cdot )$ denotes the indicator function. Then, the C-index can be estimated by

$$\begin{aligned} {\hat{C}} = m^{-1}\sum _{i, j: T_i \le T_j} I[({\hat{S}}(t|X_i) \le {\hat{S}}(t|X_j)], \end{aligned}$$

where $\hat{S}(t|X_{i})$ is the predicted survival function with covariate $X_i$ and $m = \sum _{i,j}I(T_i \le T_j)$.

The C-index value is a proportion between 0 and 1. Values near 1 indicate high model discrimination performance, whereas values near 0.5 show similarity to random prediction^20^. Note that there are various methods for calculating C-index for right-censored data. Harrell’s C-index depends on censoring distribution because they provided C-index censored information largely excluded^22^. Otherwise, Uno^23^ and Efron^21^’s approaches are censoring-independent.

#### Integrated brier score

Another criterion used to compute risk prediction is the time-dependent Brier Score (BS)^24,25^. The BS can be defined by the mean squared error between the predicted survival function $\hat{S}(t|X_{i})$ and the predictor variable $X_{i}$ and the true status $Y_{i}(t)$ at a specified time-point *t*^26^. In the presence of right censored data, the BS can be estimated by

$$\begin{aligned} {\widehat{BS}}(t|\hat{S}) = \frac{1}{M}\sum _{i\in \tilde{D}_{M}}\hat{W}_{t}(t)\{\tilde{Y}_{i}(t)-\hat{S}(t|X_{i})\}^2, \end{aligned}$$

where $\tilde{Y}_{i}(t)$ represents the observed status at time *t*. $\tilde{D}_{M}$ is the test dataset with sample size *M* and $\hat{W}_{i}(t)$ is the inverse probability of censoring weights^24^ defined by

$$\begin{aligned} \hat{W}_{i}(t) = \frac{(1-\tilde{Y}_{i}(t))\delta _{i}}{\hat{G}(T_{i}-|X_{i})}+\frac{\tilde{Y}_{i}(t)}{\hat{G}(t|X_{i})}. \end{aligned}$$

Here, $\delta _{i}$ is the event indicator, $\hat{G}(t|x)\approx P(C_{i} > t|X_{i}=x)$ is an estimate of the conditional survival function of censoring time *C* and $\hat{G}(T_{i}-|X_{i})$ denotes the estimated survival function prior to $T_{i}$ for the censoring time *C*. The estimated IBS is calculated by integration:

$$\begin{aligned} {\widehat{IBS}} = \frac{1}{max(t_{i})}\int _{0}^{max(t_{i})}{\widehat{BS}}(t|\hat{S})dt. \end{aligned}$$

Because IBS is based on the mean squared error, lower values are considered a better predictor.

#### Calibration slope

Calibration refers to the agreement between the predicted probabilities and observed number of events^27^. As described by Ambler et al.^28^, the calibration was assessed using a linear regression model to show the association between the observed and predicted values at time *t*. Thus, the regression model is defined as:

$$\begin{aligned} log\left( \frac{1-S_{i}(t|X_{i})}{S_{i}(t|X_{i})}\right) = intercept + slope\times log\left( \frac{1-\hat{S_{i}}(t|X_{i})}{\hat{S_{i}}(t|X_{i})}\right) + error, \end{aligned}$$

where $S_{i}(t|X_{i})$ and $\hat{S_{i}}(t|X_{i})$ are the true and estimated survival functions for the subject *i* at time *t*, respectively. When the model is perfectly calibrated, the estimated Calibration slope is 1. If the slope is higher than 1, the model is under-fitted. In contrast, overfitted models show a Calibration slope lower than 1, meaning that the model underestimates the probability of an event in the low-risk group and overestimates it in the high-risk group.

### Simulation study

To compare the performances of the three methods, we consider the following simulation study. We first generated time-to-event, $T_i$, based on the Cox-exponential hazards model with one event of interest and one competing risk (*i* = 1,2), considering the following form:

$$\begin{aligned} T_{i} = -\frac{\log (U_{i})}{\lambda _{i}\exp (g(X_i, \beta ))}, \end{aligned}$$

where $U_{i}$ is generated from a uniform distribution and the baseline hazards are $\lambda _{1} = 1$ and $\lambda _{2} = 2$, respectively. For the *i*th state of events, five independent predictors $X_{ij} (i=1,2; j=1,...,5)$ are generated from the standard normal distribution. For the parametric function $g(X_i, \beta ))$, we consider linear and nonlinear terms (Table 1). The true regression coefficients are set as follows:

$$\begin{aligned}{} & {} (\beta _{11},\cdots ,\beta _{110}) = (1, 0, 1, -1, 1.5, 0.6, 0.6, 0.6, 0.6, 0.6),\\{} & {} (\beta _{21},\cdots ,\beta _{210}) = (0, 2, 1.5, 1, 1, 0.3, 0.3, 0.3, 0.3, 0.3). \end{aligned}$$

**Table 1:** Summary of simulation settings.

	Parametric function for generating survival time
Scenario 1	Linear : $g(X_i; \beta ) = \sum _{j=1}^5 X_{ij}\beta _{ij}$
Scenario 2	Nonlinear : $g(X_i; \beta ) = \sum _{j=1}^5 X_{ij}\beta _{ij} + (X_{i1}\cdot X_{i2}) \beta _{i6} + X_{i1}^2 \beta _{i7} + (X_{i4}\cdot X_{i5})\beta _{i8} + X_{i3}^3 \beta _{i9} + (X_{i1} \cdot X_{i4})\beta _{i10}$

To investigate the performance of each method when the parametric function is correct or misspecified, we fitted two CS models when $g(X_i; \beta )$ is linear (CS_l) and nonlinear (CS_nl). Similarly, the FG model when $g(X_i; \beta )$ is linear (FG_l) and nonlinear (FG_nl), respectively. Note that RSF does not require the selection of the parametric function $g(X_i; \beta )$. We implemented 500 and 1000 sample sizes and split the training and test sets with a 7:3 ratio in each simulation, which was repeated with 1000 independent observations. In addition, to examine the effect of censoring rates, we consider different censoring rates (30%, 60%, 80%) for each simulation.

## Results

### Baseline characteristics

**Figure 1:**
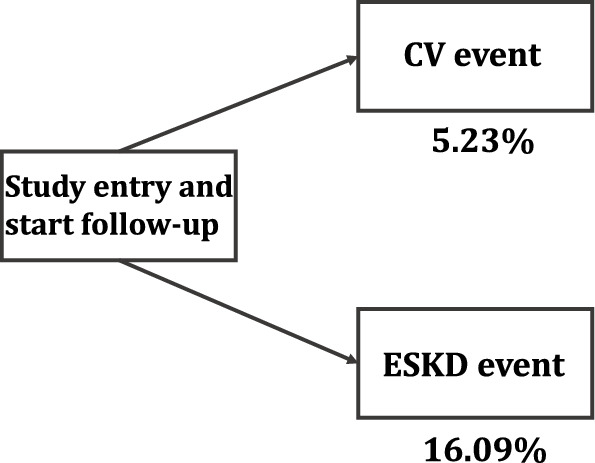
The 4-year incidence rates for CV and ESKD events in CKD subjects.

**Figure 2:**
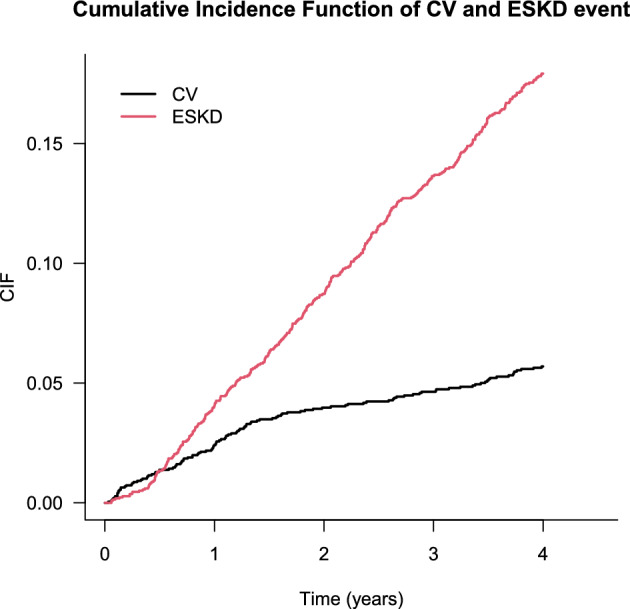
Cumulative incidence function curves for each event.

Baseline characteristics of a total of 2238 patients are shown in Table 2. Here, we considered a CV event as the event of interest. Since a CV event that occurred after the development of ESKD was not followed for practical reasons, an ESKD event can be regarded as a competing risk. We focused on the 4-year incidence rate with clinical considerations, and the incidence rates of CV and ESKD events were 117 (5.23%) and 360 (16.09%) as shown in Fig. 1. The cumulative incidence function can be defined as the expected proportion of subjects with a specific event over time^29^ alongside with Kaplan-Meier estimator without any competing risks. The 4-year cumulative incidence of CV and ESKD events in all subjects is described in Fig. 2 showing that the cumulative incidence of ESKD events exceeded that of the CV events.

**Table 2:** Baseline covariates summary in KNOW-CKD data.

Age	Mean (SD)	53.68 (12.24)
	Median (range)	55.00 (20.00–75.00)
Gender	Female	871 (38.92)
CAD	Yes	135 (6.03)
DM	Yes	754 (33.69)
CVD	Yes	348 (15.55)
Smoking	Current/former	1027 (45.89)
CKD_stage	Stage2	425 (18.99)
	Stage3a	369 (16.49)
	Stage3b	466 (20.82)
	Stage4	479 (21.40)
	Stage5	139 (6.21)
BMI	Mean (SD)	24.58 (3.40)
	Median (range)	24.40 (14.9–45.40)
HG	Mean (SD)	12.83 (2.02)
	Median (range)	12.80 (7.30–18.80)
CRP	Mean (SD)	2.05 (5.34)
	Median (range)	0.60 (0.02–68.00)
MAP	Mean (SD)	93.95 (11.68)
	Median (range)	93.33 (54.67–149.00)
LDL	Mean (SD)	96.95 (31.89)
	Median (range)	93.00 (21.00–273.00)

* SD: standard deviation; CAD: baseline coronary artery disease; DM: baseline diabetes mellitus; CVD: baseline cardiovascular disease; BMI: body mass index; HG: baseline hemoglobin; CRP: baseline high sensitivity C-reactive protein; MAP: baseline mean arterial pressure; LDL: baseline low-density lipoprotein cholesterol.

### Analysis of CKD data

The results of the two competing risk models are similar (Table 3). According to the CS model, age, gender, DM, and CVD were significantly associated with CV events; HRs were 1.04 (95% CI 1.02,1.07), 0.44 (95% CI 0.20,0.97), 2.15 (95% CI 1.28, 3.60), and 3.15 (95% CI 1.73, 5.72) respectively. Likewise, age, DM, and CVD were significantly associated with CV events in the FG model; HRs were 1.05 (95% CI 1.02, 1.07), 2.11 (95% CI 1.21, 3.66), and 3.03 (95% CI 1.68, 5.48), respectively. In addition, we evaluated the model performance by calculating the predicted probabilities and compared the results. All three results are similar, except for the Calibration slope, which is 1.167, 0.928, and 0.971, respectively (Table 4).

**Table 3:** Hazard ratios (HR) with their confidence intervals, and *p*-values in the CS and FG results.

Covariate	CS method		FG method	
	HR (95% CI)	*p*-value	HR (95% CI)	*p*-value
Age$^2$	1.04 (1.02, 1.07)	**0**.**002**	1.05 (1.02, 1.07)	${\varvec{<}}.{\textbf {001}}$
Gender (ref.male)	0.44 (0.20, 0.97)	**0**.**041**	0.48 (0.22, 1.03)	0.061
CAD	1.34 (0.71, 2.55)	0.373	1.38 (0.72, 2.63)	0.330
DM	2.15 (1.28, 3.60)	**0**.**004**	2.11 (1.21, 3.66)	**0**.**008**
CVD	3.15 (1.73, 5.72)	${\varvec{<}}.{\textbf {001}}$	3.03 (1.68, 5.48)	${\varvec{<}}.{\textbf {001}}$
Smoking	0.98 (0.54, 1.77)	0.947	1.00 (0.55, 1.83)	1.000
CKD_stage	1.03 (0.83, 1.28)	0.766	0.95 (0.75, 1.19)	0.630
BMI	0.95 (0.88, 1.03)	0.195	0.95 (0.89, 1.02)	0.160
HG	0.95 (0.82, 1.11)	0.511	0.99 (0.82, 1.02)	0.920
CRP	1.01 (0.98, 1.04)	0.700	1.01 (0.98, 1.04)	0.540
LDL	1.00 (1.00, 1.01)	0.589	1.00 (0.99, 1.01)	0.770
MAP	1.00 (0.98, 1.02)	0.946	1.00 (0.98, 1.02)	0.670

* The bold numbers represent statistically significant with 5% significance. The age is included as a square term. CAD: baseline coronary artery disease; DM: baseline diabetes mellitus; CVD: baseline cardiovascular disease; BMI: body mass index; HG: baseline hemoglobin; CRP: baseline high sensitivity C-reactive protein; MAP: baseline mean arterial pressure; LDL: baseline low-density lipoprotein cholesterol.

**Figure 3:**
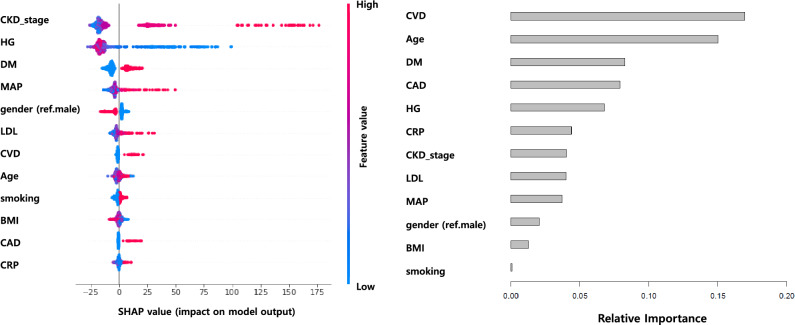
SHAP value (left) and Variable Importance (right) in RSF result.

**Table 4:** Prediction performance.

Model	C-index	IBS	Calibration slope
CS	0.776	0.031	1.167
FG	0.776	0.031	0.928
RSF	0.768	0.032	0.971

Lastly, we calculated SHapley Additive exPlanations (SHAP) values and Variable Importance to identify the contribution of the risk factors. As shown in Fig. 3, the left panel displays SHAP values meaning that the first variable on the top is the most important and the last variable on the bottom is the least important. High values of CKD_stage, MAP, and LDL positively contributed to predicting a CV event. DM and CAD were also positively associated with the prediction of CV events. On the other hand, HG and BMI have a negative relationship with predicting a CV event, and males are associated with a higher incidence of a CV event than females. The variable Importance from RSF result is also represented in the right side of Fig. 3 showing that CVD is the most influential predictive factor for the incidence of a CV event. Then, age, DM, CAD, and HG were assessed. CVD, age, and DM were important clinical factors for predicting CV events when ESKD was considered a competing risk in three results. In the case of SHapley values, the CKD stage is the most important factor in predicting CV events. Age, DM, male sex, and advanced CKD stage have been reported as risk factors for adverse CV events in previous studies.^30–32^

### Simulation results

**Figure 4:**
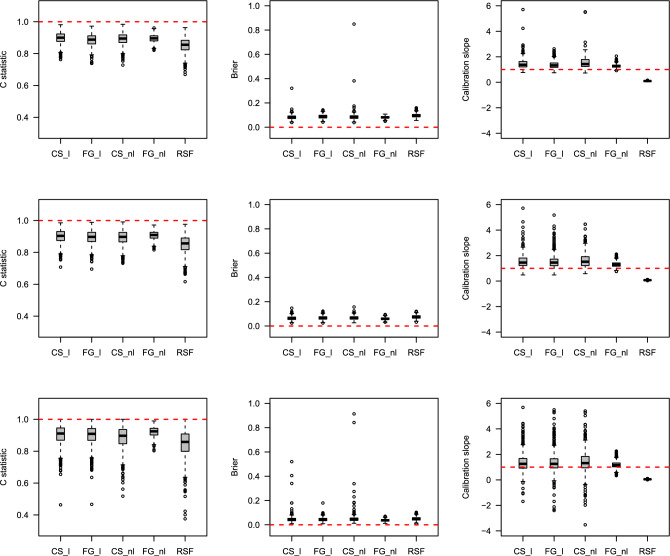
Boxplots of predicted probabilities by each method with the existence of linear effects in time-to-event with sample size n$=500$. The top, middle and bottom results represent 30%, 60%, and 80% censoring rates, respectively. The horizontal dashed line represents the optimal value in each plot.

**Figure 5:**
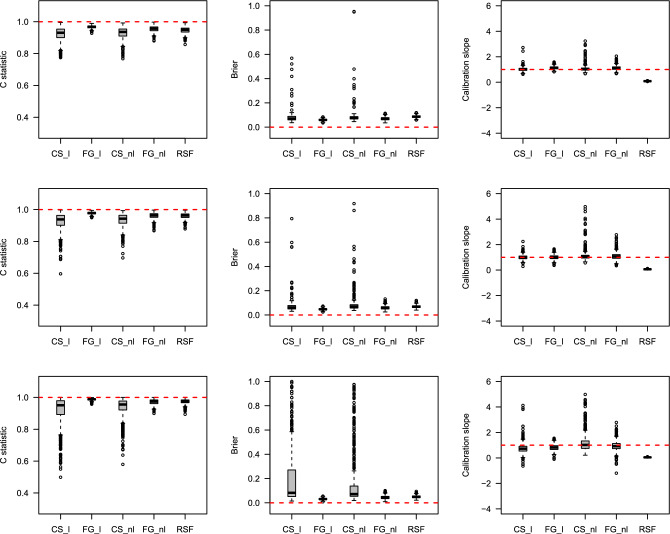
Boxplots of predicted probabilities by each method with the existence of nonlinear effects in time-to-event with sample size n$=500$. The top, middle and bottom results represent 30%, 60%, and 80% censoring rates, respectively. The horizontal dashed line represents the optimal value in each plot.

**Figure 6:**
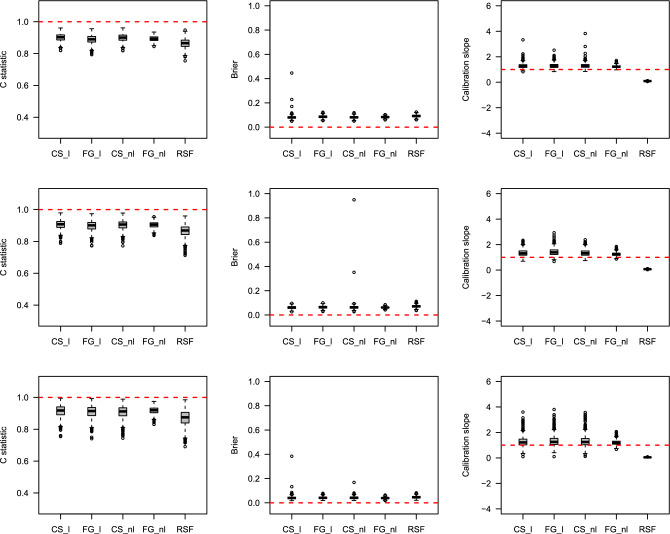
Boxplots of predicted probabilities by each method with the existence of linear effects in time-to-event with sample size n$=1000$. The top, middle and bottom results represent 30%, 60%, and 80% censoring rates, respectively. The horizontal dashed line represents the optimal value in each plot.

**Figure 7:**
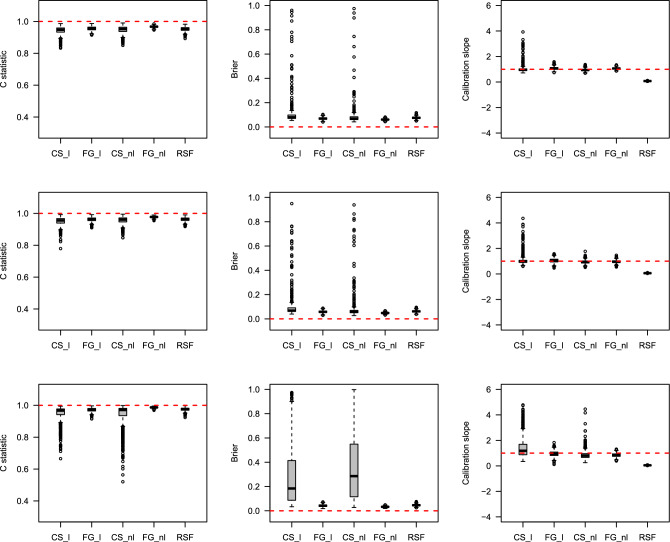
Boxplots of predicted probabilities by each method with the existence of nonlinear effects in time-to-event with sample size n$=1000$. The top, middle and bottom results represent 30%, 60%, and 80% censoring rates, respectively. The horizontal dashed line represents the optimal value in each plot.

The prediction performances were estimated using box plots through the C-index, IBS, and Calibration slope. Detailed descriptions of the three measures are provided in Methods section. Figures 4, 5, 6, 7 show the results of the prediction performance by varying the sample size ($n=500$ and 1000) and scenarios (1 and 2). In Figs. 4 and 6 (when the true $g(X_i; \beta )$ is linear), as the censoring rates increase, the three prediction measures have similar performance on average but become highly volatile. In particular, the Calibration slope revealed increased overfitting and underfitting. Because the true $g(X_i; \beta )$ is linear, the correct models CS_l and FG_l perform similarly to the models CS_nl and FG_nl models. Interestingly, RSF was slightly worse than the correct models in terms of the C-index and IBS, and its Calibration slope is lower than 1 (overfitted) in all cases. Figures 5 and 7 (when the true $g(X_i; \beta )$ is nonlinear), the misspecified models CS_l and FG_l are poorer than their correct models. Among the methods, the performance of the CS method was poorer than that of the other methods. Their C-index was much lower, the IBS was much larger, and the range of the estimated Calibration slope was too wide, indicating overfitting and underfitting. This showed that the CS model with linear and nonlinear effects in time-to-event was more susceptible as the censoring rates increased. In summary, the performance of each method deteriorated as the censoring rate increased. However, if the conditions are the same, the results with larger sample sizes show a better and more stable performance. The CS approach is more sensitive to censoring rates than the FG and RSF methods.

## Discussion

In this study, we analyzed a multicentre cohort study of chronic kidney disease (KNOW-CKD) data based on the competing risks framework. We investigated the association between clinical risk factors and renal dysfunction and compared their estimates, predicted abilities, and feature importance. As the previous studies have shown that age, male sex, diabetes, and known vascular disease are risk factors for the 10-year development of CV diseases in the general population^33^, our results from the CKD population were also similar. In the CS result, age, gender, DM, and CVD variables were significant. In the FG result, age, DM, and CVD were significant but gender was marginally significant. Although RSF cannot directly provide the *p*-values for significance, the variable importance was calculated. Analysis from Chronic Renal Insufficiency Cohort (CRIC) with advanced CKD showed increased age, diabetes, elevated blood pressure, and any CVD history are significant risk factors for CV events^34^. Other studies with KNOW-CKD subjects also reported that male sex, diabetes, and increased CKD stage are risk factors for CV events^31,35^.

In real data analysis, two conventional methods (CS, FG) and the RSF method showed similar results in terms of the significance of risk factors and prediction. Although all methods select similar variables as the relevant features and have similar prediction performances, they all have good predictive power. Therefore, we believe that the current model with linear terms is reasonable to fit our data. Thus, we have conducted simulation studies by considering linear and non-linear terms. The performance of each method decreases as the censoring rate increase. These results show that more sophisticated methods must be considered when developing prediction models with few events. Based on the simulation results when the non-linear model is true, the RSF method was more robust. Therefore, the RSF method is recommended when the prediction performances are similar.

We only used the baseline covariates as the feature variables to analyze the competing risks data. Our current analysis provided ease of computation and straightforward prediction of survival time. Because the KNOW-CKD study is longitudinal, some relevant covariates related to CKD patients can be time-dependent. Extension to a model where the time-dependent covariates or time-varying coefficients are considered would be interesting future work.

## Data Availability

The datasets used and/or analyzed during the current study are available from the corresponding author upon reasonable request.

## References

[1] Cox DR (1972). Regression models and life-tables. J. Roy. Stat. Soc.: Ser. B (Methodol.).

[2] Cox DR (1975). Partial likelihood. Biometrika.

[3] Austin PC, Lee DS, Fine JP (2016). Introduction to the analysis of survival data in the presence of competing risks. Circulation.

[4] Lau B, Cole SR, Gange SJ (2009). Competing risk regression models for epidemiologic data. Am. J. Epidemiol..

[5] Putter H, Fiocco M, Geskus RB (2007). Tutorial in biostatistics: Competing risks and multi-state models. Stat. Med..

[6] Andersen, P. K., Borgan, Ø., Hjort, N. L., Arjas, E., Stene, J., & Aalen, O. Counting process models for life history data: A review [with discussion and reply]. Scand. J. Stati., 97-158.

[7] Fine JP, Gray RJ (1999). Proportional hazards model for the subdistribution of competing risks. J. Am. Stat. Assoc..

[8] Ishwaran H, Gerds TA, Kogalur UB, Moore RD, Gange SJ, Lau BM (2014). Random survival forests for competing risks. Biostatistics.

[9] Kayes OJ, Loddo M, Patel N, Patel P, Minhas S, Ambler G, Williams GH (2009). DNA replication licensing factors and aneuploidy are linked to tumor cell cycle state and clinical outcomes in penile carcinoma. Clin. Cancer Res..

[10] Oh KH, Park SK, Park HC, Chin HJ, Chae DW, Choi KH, Ahn C (2014). KNOW-CKD (KoreaN cohort study for outcome in patients with chronic kidney disease): Design and methods. BMC Nephrol..

[11] Kang E, Han M, Kim H, Park SK, Lee J, Hyun YY, Oh KH (2017). Baseline general characteristics of the Korean chronic kidney disease: Report from the KoreaN cohort study for outcomes in patients with chronic kidney disease (KNOW-CKD). J. Korean Med. Sci..

[12] Kim, H. J., Ryu, H., Kang, E., Kang, M., Han, M., Song, S. H., & Oh, K. H. (2021). Metabolic acidosis is an independent risk factor of renal progression in Korean chronic kidney disease patients with CKD: the KNOW-CKD Study results. Front. Med. **8**. 10.3389/fmed.2021.707588 PMC8358180 34395482

[13] Zhang Z. (2017). Survival analysis in the presence of competing risks. Annals Translat. Med., **5**(3). 10.21037/atm.2016.08.62 PMC5326634 28251126

[14] Ishwaran H, Kogalur UB, Blackstone EH, Lauer MS (2008). Random survival forests. Annals Appl. Stat..

[15] Breiman L (2001). Random forests. Mach. Learn..

[16] Shapley L, Kuhn H, Tucker A (1953). Value for n-person Games. Contributions to the Theory of Games II.

[17] Lundberg, S. M., & Lee, S. I. (2017). Unified approach for interpreting model predictions. *Adv. Neural Inform. Process. Syst.* **30**.

[18] Moncada-Torres A, van Maaren MC, Hendriks MP, Siesling S, Geleijnse G (2021). Explainable machine learning can outperform Cox regression predictions and provide insight into breast cancer survival. Sci. Rep..

[19] Harrell JE, Lee KL, Califf RM, Pryor DB, Rosati RA (1984). Regression modeling strategies for improved prognostic prediction. Stat. Med..

[20] Harrell FE, Lee KL, Mark DB (1996). Multivariable prognostic models: Issues in developing models, evaluating assumptions and adequacy, and measuring and reducing errors. Stat. Med..

[21] Brentnall AR, Cuzick J (2018). Use of the concordance index for predictors of censored survival data. Stat. Methods Med. Res..

[22] Harrell FE, Califf RM, Pryor DB, Lee KL, Rosati RA (1982). Evaluating the yield of medical tests. JAMA.

[23] Uno H, Cai T, Pencina MJ, D’Agostino RB, Wei LJ (2011). On the C-statistics for evaluating overall adequacy of risk prediction procedures with censored survival data. Stat. Med..

[24] Graf E, Schmoor C, Sauerbrei W, Schumacher M (1999). Assessment and comparison of prognostic classification schemes for survival data. Stat. Med..

[25] Gerds TA, Schumacher M (2006). Consistent estimation of the expected Brier score in general survival models with right-censored event times. Biom. J..

[26] Mogensen UB, Ishwaran H, Gerds TA (2012). Evaluation of random forests for survival analysis using prediction error curves. J. Stat. Softw..

[27] Van Calster B, Nieboer D, Vergouwe Y, De Cock B, Pencina MJ, Steyerberg EW (2016). A calibration hierarchy for risk models was defined from utopia to the empirical data. J. Clin. Epidemiol..

[28] Ambler G, Seaman S, Omar RZ (2012). An evaluation of penalised survival methods for developing prognostic models with rare events. Stat. Med..

[29] Latouche A, Allignol A, Beyersmann J, Labopin M, Fine JP (2013). A competing risk analysis should report the results for all cause-specific hazards and cumulative incidence functions. J. Clin. Epidemiol..

[30] Toth-Manikowski SM, Yang W, Appel L, Chen J, Deo R, Frydrych A, Unruh ML (2021). Sex Differences in Cardiovascular Outcomes in CKD: Findings From the CRIC Study. Am. J. Kidney Dis..

[31] Ryu H, Kim J, Kang E, Hong Y, Chae DW, Choi KH, Oh KH (2021). Incidence of cardiovascular events and mortality in Korean patients with chronic kidney disease. Sci. Rep..

[32] Matsushita, K., Ballew, S. H., Wang, A. Y. M., Kalyesubula, R., Schaeffner, E., & Agarwal, R. (2022). Epidemiology and risk of cardiovascular disease in patients with chronic kidney disease. *Nat. Rev. Nephrol.*, 1-12. 10.1038/s41581-022-00616-6 36104509

[33] D’Agostino RB, Vasan RS, Pencina MJ, Wolf PA, Cobain M, Massaro JM, Kannel WB (2008). General cardiovascular risk profile for use in primary care: The Framingham Heart Study. Circulation.

[34] Grams ME, Yang W, Rebholz CM, Wang X, Porter AC, Inker LA, Townsend RR (2017). Risks of adverse events in advanced CKD: the chronic renal insufficiency cohort (CRIC) study. Am. J. Kidney Dis..

[35] Jung CY, Heo GY, Park JT, Joo YS, Kim HW, Lim H, Han SH (2021). Sex disparities and adverse cardiovascular and kidney outcomes in patients with chronic kidney disease: Results from the KNOW-CKD. Clin. Res. Cardiol..

