# Author Correction: Comparing predictions among competing risks models with rare events: application to KNOW-CKD study—a multicentre cohort study of chronic kidney disease

**DOI:** 10.1038/s41598-023-42772-0

**Published:** 2023-09-19

**Authors:** Jayoun Kim, Soohyeon Lee, Ji Hye Kim, Dha Woon Im, Donghwan Lee, Kook-Hwan Oh

**Affiliations:** 1 Medical Research Collaborating Center, Seoul National University Hospital, Seoul, Republic of Korea; 2 Department of Statistics, Ewha Womans University, Seoul, Republic of Korea; 3 Department of Internal Medicine, Chungbuk National University Hospital, Cheongju, Korea; 4 Department of Internal Medicine, Seoul National University Hospital, Seoul, Republic of Korea

Correction to: *Scientific Reports* https://doi.org/10.1038/s41598-023-40570-2, published online 16 August 2023

The original version of this Article contained an error in the name of Donghwan Lee, which was incorrectly given as Dongwhan Lee.

The original Article has been corrected.

